# Bacterial Isolates from Avocado Orchards with Different Agronomic Management Systems with Potential for Promoting Plant Growth in Tomate and Phytopathogen Control

**DOI:** 10.3390/microorganisms13091974

**Published:** 2025-08-23

**Authors:** Adilene Velázquez-Medina, Evangelina Esmeralda Quiñones-Aguilar, Ernestina Gutiérrez-Vázquez, Nuria Gómez-Dorantes, Gabriel Rincón-Enríquez, Luis López-Pérez

**Affiliations:** 1 Instituto de Investigaciones Agropecuarias y Forestales (IIAF), Universidad Michoacana de San Nicolás de Hidalgo (UMSNH), Km 9.5 Carr. Morelia Zinapécuaro, Tarímbaro 58880, Michoacán, Mexico; 1129881h@umich.mx (A.V.-M.); ernestina.gutierrez@umich.mx (E.G.-V.); nuria.gomez@umich.mx (N.G.-D.); 2 Laboratorio de Fitopatología, Unidad de Biotecnología Vegetal, Centro de Investigación y Asistencia en Tecnología y Diseño del Estado de Jalisco A.C. (CIATEJ), Camino Arenero 1227, El Bajío del Arenal, Zapopan 45019, Jalisco, Mexico; equinones@ciatej.mx (E.E.Q.-A.); grincon@ciatej.mx (G.R.-E.)

**Keywords:** conventional management, organic management, bacterial diversity, rhizosphere, antagonism, plant growth-promoting bacteria, biocontrol

## Abstract

The bacterial diversity of soils cultivated with avocado (*Persea americana* M.) is influenced by different factors, perhaps the most decisive being the type of agronomic management used by farmers. In conventional agronomic management (CM), high doses of agrochemicals are applied, in contrast to organic agronomic management (OM), where organic fertilizers are used. This alters the diversity and abundance of soil microorganism populations, which in turn affects crop health. This study aimed to isolate and morphologically characterize rhizospheric bacteria from avocado trees under different agronomic management systems (CM and OM). For the bacterial isolates, their ability to promote plant growth in vitro was determined through biochemical tests for phosphorus and calcium solubilization and nitrogen fixation. In addition, their in vivo effect on tomato (*S. lycopersicum*) growth was evaluated, and their antagonistic capacity against *Fusarium* sp. was assessed. The results showed differences in the quantity, diversity, and morphologies of bacterial isolates depending on the type of agronomic management. A higher Shannon diversity index was found in OM (2.44) compared to CM (1.75). A total of 35 bacterial isolates were obtained from both management types. A greater number of isolates from OM soils exhibited in vitro PGP activity; notably, eight isolates from OM plots showed phosphate-solubilizing activity, compared to only one from CM plots. Furthermore, although all isolates demonstrated nitrogen fixing capacity, those from OM orchards produced significantly higher nitrate levels than the control (*Azospirillum vinelandii*). On the other hand, inoculation of tomato plants with bacterial isolates from OM soils increased plant height, root length, and total fresh and dry biomass compared to isolates from CM soils. Likewise, OM isolates exhibited greater antagonistic activity against *Fusarium* sp. These findings demonstrate the impact of agronomic management on soil bacterial populations and its effect on plant growth and protection against pathogens.

## 1. Introduction

Avocado cultivation requires nutrient-rich soils to support high productivity and plant health [1]. To meet these conditions, farmers often apply large amounts of agrochemicals. However, prolonged use of these products alters soil microorganism populations (mainly bacteria) and causes environmental harm such as soil and groundwater contamination due to nutrient leaching and poisoning issues among farmers [2]. Agricultural practices are known to affect soil bacterial populations, which can limit agroecosystem productivity. Faced with the challenge of sustainably increasing avocado crop yields, the use of plant growth-promoting bacteria (PGPB) is a promising strategy to reduce agrochemical dependency [3]. PGPB mainly inhabit the rhizosphere, the area surrounding plant roots, and some form crucial symbiotic relationships with the host plant, enhancing productivity and plant health under various cultivation and environmental conditions [4,5]. In Mexico, particularly in the avocado-producing region of Michoacán, two main agricultural management systems are used: conventional and organic. The key difference lies in the use of agrochemicals in CM versus organic inputs in OM for nutrient supply and disease management. These practices significantly alter the physical, chemical, and microbiological properties of the soil [6,7]. In CM, the heavy use of agrochemicals (fungicides, bactericides such as Agry-Gent plus 800: gentamicin sulfate + oxytetracycline hydrochloride and Bactermicina 500: streptomycin + oxytetracycline, and fertilizers) disrupts the biological fertility of the soil by reducing rhizospheric bacterial populations. In addition to the problems associated with the persistence of compounds such as chlorothalonil, imidacloprid, and glyphosate, these are also widely used in CM and are resistant to decomposition and can persist and accumulate in soil and water, representing a serious environmental threat [8].

Likewise, bacteria play essential roles in nutrient cycling, carbon balance, transformation of organic to inorganic compounds, and plant health, among others [9]. In contrast, OM employs organic amendments (manure from various sources, composts, vermicompost, leachates), which, in addition to improving soil nutrition and physical properties, introduce diverse bacterial populations that may promote plant growth and act antagonistically against phytopathogens helping preserve the soil and increase crop productivity [10,11,12].

The variation in bacterial diversity due to agricultural practices has been widely studied; Acharya et al. [13] reported a Shannon diversity index (SDI) of 6.5 in organic pasture soils, noting that iron, magnesium, carbon, and nitrogen contents positively correlated with bacterial diversity. Bebber and Richards [14] reported higher diversity in organically managed soils compared to those managed conventionally with chemical fertilizers (NPK), with the organic system showing 1.5 times greater diversity. Despite CM altering bacterial diversity, it is undeniable that PGPB can still be found in such systems. For instance, Melo et al. [15] isolated bacteria from the rhizosphere of papaya (*Carica papaya*) grown under both CM and OM, finding phosphate-solubilizing bacteria (PSB) of the genera *Burkholderia, Klebsiella*, and *Leclercia* in OM, while *Enterobacter* sp. (CM) and *Klebsiella* (OM) not only solubilized phosphates but also produced siderophores and inhibited the mycelial growth of phytopathogens like *Botrytis cinerea* and *Fusarium culmorum*. Teherán-Sierra et al. [16] studied bacterial isolates from soil, roots, stems, and leaves of sugarcane grown under both systems and identified 84 isolates in OM and 76 in CM. Seventeen strains of actinobacteria, bacteroidetes, firmicutes, and proteobacteria were phosphate-solubilizing, produced auxins and siderophores, and positively affected *Cynodon dactylon* germination. Corrales-Lozada [17] isolated rhizospheric bacteria from purslane (*Portulaca oleracea*) under CM, with 88.9% fixing atmospheric nitrogen, mainly of the genus *Azospirillum*, which produced 64 ppm of ammonium (NH_4_^+^) in vitro. Additionally, 96% of the isolates solubilized phosphate, with *Burkholderia* being the predominant genus, showing 64.9 ppm. Sherpa et al. [18] isolated bacteria from organically managed pea (*Pisum sativum*) soils and identified *Bacillus cereus* and *Variovorax paradoxus* with in vitro phosphate solubilization capacity. In avocado, Anaya et al. [19] isolated rhizospheric bacteria from conventionally managed plots, identifying *Burkholderia cepacia*, *Enterobacter cloacae*, *Pseudomonas aeruginosa*, *Bacillus cereus*, and *Bacillus megaterium*, all showing phosphate solubilization and nitrogen fixation capacities in vitro. Tomato (*Solanum lycopersicum*) production in Mexico is of great importance; currently the country is the ninth largest producer worldwide and the main exporter to the United States, Japan, and several European countries. To meet high demand, farmers often apply large quantities of chemical fertilizers, resulting in environmental harm such as nitrate leaching, soil acidification, groundwater contamination, biodiversity loss, etc. [20]. Therefore, finding sustainable alternatives for tomato production is essential to transition toward a more sustainable agricultural system. Given this, the present study focused on the isolation and morphological characterization of rhizospheric bacteria from productive-stage avocado trees in orchards under different agronomic practices (CM and OM) and on the evaluation of their plant growth-promoting capacity through in vitro assays for phosphorus and calcium solubilization and biological nitrogen fixation, as well as their effectiveness in promoting plant growth in tomato (*S. lycopersicum*) plants and finally their antagonistic activity against *Fusarium* sp.

## 2. Materials and Methods

### 2.1. Study Areas and Soil Sampling

The avocado plots (*Persea americana* M.), under conventional management (CM), were located in the avocado-producing region of the municipality of Tingambato, Michoacán, at coordinates 19.598261, −101.870625 and 19.597336, −101.868730. This region has a maximum temperature of 26 °C and a minimum of 12 °C, with 70% humidity. These plots practice agronomic management including pH regulation with applications of calcium hydroxide Ca(OH)_2_, chemical fertilization with a 20:30:10 (N:P:K) ratio, and the use of synthetic insecticides and fungicides. The organically managed plots (OM), were located in the municipality of Acuitzio del Canje, Michoacán, at coordinates 19.434004, −101.277925. In these plots, pH is regulated using dolomite, and organic amendments such as diatomaceous earth, feedlot cattle manure, phosphate rock, vermicompost, and other organic products like arbuscular mycorrhizal fungi are applied. These orchards, being organically certified, do not use agrochemicals. Rhizosphere sampling was conducted during the rainy season. Four trees were selected per plot, for a total of 16 sampled trees. Approximately 1 kg of soil was collected from the drip and rhizosphere zones of each tree, and all samples were then mixed and homogenized to obtain a composite sample per plot and management type. The composite samples soil from each plot were placed in plastic bags and transported to the Plant Ecophysiology Laboratory of the Instituto de Investigaciones Agropecuarias y Forestales de la Universidad Michoacana de San Nicolás de Hidalgo for processing. The total composite soil samples were air-dried, and a mix of CM and OM samples was sent to the Soil Fertility Laboratory of the Instituto Nacional de Investigaciones Forestales, Agrícolas y Pecuarias, in Celaya Guanajuato, México, for physicochemical characterization.

### 2.2. Isolation, Morphological Characterization, and Shannon Diversity Index of Bacterial Isolates

Serial dilutions up to 10^6^ were prepared from 1 g from each of the subsamples that made up the composite sample for each plot from orchards under different management systems, using 9 mL of sterile deionized water. The dilutions were inoculated on Petri dishes containing nutrient agar using the spread plate method with a Digralsky loop and incubated at 32 °C for 36 h. Colonies with distinct morphological characteristics were selected and purified following the methodology described by Muñoz et al. [21]. Each isolate was described macroscopically (size, shape, edge, transparency, brightness, color, texture, elevation, consistency), and Gram staining was performed using a HYCEL differential bacterial staining kit, following Alcarraz et al. [22]. Bacterial diversity was calculated using the Shannon Diversity Index [23].

### 2.3. Plant Growth-Promoting Traits of Bacterial Isolates

#### 2.3.1. Phosphate Solubilization

Bacterial isolates were evaluated in NBRIP medium (g·L^−1^: 10 glucose, 5 Ca_3_(PO_4_)_2_, 0.25 MgSO_4_·7H_2_O, 0.2 KCl, 5 MgCl_2_·6H_2_O, 0.1 (NH_4_)_2_SO_4_, 17 bacteriological agar; pH adjusted to 7; Nautiyal [24]. Isolates were incubated at 32 °C for 15 days; those producing a transparent halo or clear zone around the colony were assessed for the solubilization index using the formula proposed by Corrales-Ramírez et al. [25]: SI = [Colony diameter (mm) + Halo diameter (mm)]/Colony diameter (mm)

#### 2.3.2. Acid and Alkaline Phosphatase Activity

Acid and alkaline phosphatase activities were determined using the methodology proposed by Gómez-Guiñán [26]. A volume of 5 mL from each positive bacterial isolate was diluted in 45 mL of sterile distilled water, then transferred to 20 mL of universal buffer at pH 5.5 (2.5 g CH_3_COONa and 1.8 g CH_3_COOH in 100 mL) and pH 9.0 (1.34 g NH_4_Cl and 1.8 mL NaOH in 100 mL) for acid and alkaline phosphatase activity, respectively. The mixtures were homogenized at 800 rpm for 30 s, 3 mL of the suspension was recovered, and 1 mL of 0.025 M p-nitrophenyl phosphate was added. Samples were incubated at 37 °C for 3 h, centrifuged at 2000 rpm for 10 min, and 0.5 mL of the supernatant was mixed with 4.5 mL of 0.5 M NaOH. p-Nitrophenyl phosphate released was quantified by measuring absorbance at 400 nm. A 20 µg·mL^−1^ standard solution of p-nitrophenyl phosphate was prepared, and a calibration curve was constructed by linear regression analysis [27].

#### 2.3.3. Calcium Solubilization

Pikovskaya medium [(PVK) g·L^−1^: 10 glucose, 0.5 (NH_4_)_2_SO_4_, 2.5 CaCO_3_, 0.2 NaCl, 0.1 MgSO_4_, 0.2 KCl, 0.5 yeast extract, 0.1 MnSO_4_, and 20 g bacteriological agar; pH 7] was used. Isolates were incubated at 32 °C for 8 days. A clear zone or transparent halo around the colony indicated calcium solubilization, which was measured in millimeters [23]. This data was used to calculate the solubilization index as described by Corrales-Ramírez et al. [25].

#### 2.3.4. Biological Nitrogen Fixation

Nitrogen fixation was evaluated in semi-solid NFB medium [g·L^−1^: 5 malic acid, 4 KOH, 0.5 K_2_HPO_4_, 0.02 NaCl, 0.01 CaCl_2_, 0.05 FeSO_4_, 2 mL bromothymol blue, 2 mL micronutrient solution (0.2 Na_2_MoO_4_·2H_2_O, 0.235 MnSO_4_·H_2_O, 0.28 H_3_BO_3_, 0.008 CuSO_4_·5H_2_O, 0.024 ZnSO_4_·7H_2_O), and 8 g agar; pH adjusted to 7] [28]. Isolates were inoculated into NFB medium and incubated for 15 days at 30 °C. A color change from yellow green to blue confirmed qualitative nitrogen fixation. Liquid NFB medium was then prepared to quantify atmospheric nitrogen fixed as nitrate. The medium was filtered and nitrate concentration measured with a portable ion meter (Laquatwin NO3 11).

Positive controls used included *Bacillus thuringiensis* strain BT306 for phosphate and calcium solubilization and phosphatase activity and *Azospirillum vinelandii* strain BV696 for nitrogen fixation.

### 2.4. Experiment to Evaluate the Effect of Bacterial Isolates on Tomato Plant Growth

To determine the biological effectiveness of bacterial isolates as plant growth promoters, a completely randomized experimental design was used to evaluate each isolate obtained from differently managed orchards, along with a non-inoculated control. A total of 35 treatments (isolates) were evaluated with 12 replicates.

#### Establishment and Inoculation

Seeds of *S. lycopersicum* (Saladet variety) were germinated in sterile vermiculite. Once germinated, seedlings were transplanted to germination trays containing a sterile 50:20:30 *v*/*v* mix of soil, sand, and vermiculite. Throughout the experiment, seedlings were irrigated with deionized water and kept under greenhouse conditions. Inoculants were prepared by suspending a bacterial colony in nutrient broth (MCD-LAB-7142) and incubating for 18 h at 32 °C. The optical density at 600 nm was measured for each culture. Seedlings were inoculated at the base of the stem with 1.5 mL of inoculum at a concentration of 1 × 10^8^ CFU·mL^−1^ at the time of transplant and every eight days for one month [29]. After 30 days of growth, plant height (PH), root length (RL), and total fresh and dry biomass (TFB, TDB) were evaluated.

### 2.5. Experiment to Assess Antagonistic Activity of Isolates Against Fusarium sp.

For the antagonism test against *Fusarium* sp., a completely randomized experimental design was used. The study factor was the origin of the isolate, and each of the 35 isolates was included, with an additional positive control treatment using *Bacillus subtilis* strain BV143, known for its antagonistic activity against *Fusarium*. Bacterial isolates were activated on nutrient agar (MCD-LAB-7142), and *Fusarium* sp. was grown on PDA agar (MCD-LAB-7041) containing 4 g·L^−1^ potato extract, 20 g·L^−1^ dextrose, 15 g·L^−1^ agar, pH 6.5 [30]. In vitro inhibition tests of mycelial growth of the pathogen were conducted using dual culture confrontation assays with slight modifications, as described by Trinidad-Cruz et al. [31]. Growth inhibition was calculated following Moreno-Limón et al. [32] and expressed as percentage.

### 2.6. Statistical Analysis of Experimental Data

Normality and homoscedasticity of the response variables were verified. Data were analyzed by analysis of variance (ANOVA) and mean comparisons using Tukey or Dunnett tests (for treatments with a control). All analyses were performed at a 5% significance level (*p* ≤ 0.05) using the Statistical Minitab v. 22 software.

## 3. Results

### 3.1. Physicochemical Characteristics of Soils and Bacterial Diversity

In the conventionally managed (CM) soils, the soil texture was classified as sandy clay loam, with a bulk density of 0.85 g·cm^−3^, a pH of 5.94, and 5.81% organic matter. These soils also exhibited high levels of nitrogen and potassium (Table 1). From these soils, 15 bacterial isolates were obtained, identified as IBC-1 to IBC-15, and a Shannon diversity index (SDI) of 1.75 was recorded. In contrast, the organically managed (OM) soils had a sandy loam texture, a pH of 7.08, and 10.23% organic matter, with high levels of phosphorus, calcium, magnesium, sodium, and zinc (Table 1). From these soils, 20 bacterial isolates were obtained, identified as IBO-1 to IBO-20, and a statistically different (*p* ≤ 0.05) SDI of 2.44 was recorded compared to the SDI of CM soils.

### 3.2. Morphological Characteristics of Bacterial Isolates

The macroscopic and microscopic morphological characteristics of the bacterial isolates from the different orchards varied, and greater morphological diversity was observed in the CM orchards. For macroscopic traits, the colony margins of bacterial isolates from CM orchards were wavy, while those from OM orchards were filamentous and lobate. In terms of elevation, colonies from CM isolates were convex, whereas those from OM orchards were either raised or flat. Microscopically, the bacterial isolates from CM orchards appeared as long bacilli with spores located at initial, central, or terminal positions. In contrast, isolates from OM orchards showed long, short, and wide bacilli, some with terminal spores. Regarding Gram staining, eight Gram-negative and seven Gram-positive isolates were recorded in CM orchards, while in OM orchards, 10 Gram-positive and 10 Gram-negative isolates were found (Table 2).

### 3.3. Plant Growth-Promoting Characteristics of Bacterial Isolates

#### 3.3.1. Phosphate Solubilization

Eight isolates capable of solubilizing phosphorus were recorded in the OM (organically managed) orchards, while only one was found in the CM (conventionally managed) orchards. The bacterial isolate labeled IBO-17 showed the highest solubilization index (PSI) with a value of 4.31, followed by IBO-13 (3.5 PSI) and IBO-14 (3.22 PSI), values that were statistically higher (Dunnett, *p* ≤ 0.05) than the 2.46 PSI of *B. thuringiensis* (positive control). Meanwhile, the isolates IBO-19, IBO-20, IBO-18, IBO-12, and IBO-10 recorded PSI values of 2.95, 2.91, 2.74, 2.41, and 2.14, respectively; these results were statistically similar to *B. thuringiensis* (Table 3).

#### 3.3.2. Acid and Alkaline Phosphatase Activity

Acid (APA) and alkaline phosphatase activity (ALPA) were measured in only nine bacterial isolates that solubilized phosphate. The isolate IBC-3 from CM soils showed the highest APA with 18.57 µg mL^−1^ of p-nitrophenyl phosphate (PNP), followed by IBO-13 from OM soils with 15.87 µg mL^−1^ PNP, and IBO-17 with 4.31 PSI released 14.61 µg mL^−1^ PNP. These values were statistically different (Dunnett, *p* ≤ 0.05) from the 10.22 µg mL^−1^ PNP of *B. thuringiensis*, indicating a higher number of APA-producing isolates in OM soils. Regarding ALPA, isolates IBO-13 and IBO-18 recorded 13.76 µg mL^−1^ and 14.68 µg mL^−1^ of PNP, respectively—statistically different (Dunnett, *p* ≤ 0.05) from the 3.95 µg mL^−1^ PNP measured in *B. thuringiensis* (Table 3).

#### 3.3.3. Calcium Solubilization

Only eight bacterial isolates (23% of the total) showed calcium solubilization ability: two from CM orchards (IBC-1 and IBC-2) and six from OM orchards (IBO-9, IBO-10, IBO-13, IBO-17, IBO-18, and IBO-20). The isolate IBO-9 recorded the highest calcium solubilization index (CSI) with 6.33, followed by IBC-6 with 3.05 CSI. These values were statistically different (Dunnett, *p* ≤ 0.05) from the 2.93 CSI of *B. thuringiensis*. Meanwhile, IBC-1 showed a CSI of 2.77, and the isolates from OM soils such as IBO-13 recorded 2.83 CSI, followed by 2.66, 2.21, and 1.96 CSI from IBO-18, IBO-20, and IBO-10, respectively. These values were statistically similar (Dunnett, *p* ≤ 0.05) to *B. thuringiensis* (Table 3).

#### 3.3.4. Biological Nitrogen Fixation

All isolates obtained from the orchards demonstrated nitrogen fixation activity. Of the 20 bacterial isolates from OM orchards, eleven (55%): IBO-4, IBO-5, IBO-6, IBO-7, IBO-9, IBO-12, IBO-13, IBO-15, IBO-17, IBO-18, and IBO-19, showed nitrate levels that were statistically higher (Dunnett, *p* ≤ 0.05) than the positive control *A. vinelandii*, which fixed only 62.25 ppm of NO_3_^−^. Among them, IBO-13 stood out by fixing 100 ppm more than the positive control. In contrast, all isolates from CM orchards recorded nitrate values statistically equal (Dunnett, *p* ≤ 0.05) to those fixed by the positive control *A. vinelandii*, with an average of 68.5 ppm of nitrate (Table 3).

### 3.4. Biological Effectiveness of Bacterial Isolates as Plant Growth Promoters in Tomato Plants (Solanum lycopersicum)

The different bacterial isolates obtained from orchards under different management practices showed significant effects on the growth of tomato plants (Table 4). The isolates obtained from OM orchards exhibited the highest values in all evaluated growth variables. Isolates IBO-11 and IBO-12 recorded 50% greater plant height compared to the uninoculated control plants; however, only IBO-12 demonstrated in vitro plant growth-promoting activity. Regarding root length, isolates IBO-15 and IBO-18 showed increases of 61%, and among them, only IBO-15 exhibited activity in all in vitro plant growth-promoting tests. Concerning total fresh biomass, isolates IBO-10, IBO-11, and IBO-17 showed a 300% increase over the uninoculated control. Notably, isolate IBO-11 did not exhibit any activity in the in vitro assays (Table 4). For total dry biomass, isolates IBO-11, IBO-12, and IBO-14 showed increases similar to those observed in total fresh biomass. Among the isolates from CM orchards, the most outstanding were IBC-15, which recorded a 100% increase in plant height, and IBC-4 and IBC-1, which also showed a 100% increase in root length. Isolate IBC-15 showed a 200% increase in total fresh biomass, and isolates IBC-1 and IBC-15 showed significant increases in total dry biomass compared to the uninoculated control. It is noteworthy that among these isolates, only IBC-1 demonstrated in vitro plant growth-promoting activity.

### 3.5. Biological Effectiveness of the Antagonistic Activity of Bacterial Isolates Against Fusarium sp.

The in vitro antagonistic activity of bacterial isolates obtained from orchards under different management practices against *Fusarium* sp. is presented in Table 5. Of the fifteen isolates from the CM orchards, only eight (53%) showed antagonistic activity against the pathogen *Fusarium* sp., which was statistically similar to the positive control *B. subtilis*, achieving mycelial growth inhibition percentages greater than 80%. In contrast, of the 20 isolates from OM orchards, only five (25%) exhibited antagonism against *Fusarium* sp., and their inhibition values were statistically equal to those recorded by *B. subtilis*. Notably, isolate ABO-4 recorded the lowest inhibition value, which was statistically lower (*p* ≤ 0.05, Dunnett) than that obtained by *B. subtilis*.

## 4. Discussion

In this study, 35 bacterial isolates were obtained from avocado orchards under two different management types (conventional and organic). The physicochemical characteristics of the soil plots differed, primarily due to the spatial variability in the orchards [33] but also mainly because of the type of orchard management [34]. A higher nutrient content and neutral pH were found in organically managed orchards, which can be attributed to the frequent addition of organic amendments by producers in this management system [35]. A total of 20 bacteria were isolated from organically managed orchards, five more than from conventional ones. No distinctive morphological characteristics were observed according to the management type; however, a greater diversity was found in the organic orchards. There are reports indicating that agricultural practices influence the diversity of soil microbial populations [36], and accordingly, the diversity changes [37]. Jost and González-Oreja [38] reported that greater bacterial diversity may be due to a higher organic matter content in these soils, as well as higher nutrient levels and neutral pH [34], as found in the organically managed orchards (Table 1). These characteristics would create a more favorable environment for greater bacterial diversity in these soils. Additionally, nutrient-rich soils have a greater capacity to sustain diverse bacterial populations [39,40]. Since OM orchards apply amendments such as manures, composts, and vermicompost, this likely increased the available carbon for bacterial reproduction and boosted bacterial populations in the soil [41,42,43].

In conventional orchards, the constant use of agrochemicals reduces bacterial diversity [44] and acidifies the soil [45], as was found in this study. Furthermore, concentrations of some nutrients like calcium, phosphorus, and zinc were lower compared to organic orchards. These elements are also essential for microorganisms, which could be affecting their abundance and diversity [46]. The loss of microbial diversity in the soil can be detrimental to soil health, as it has been reported that both fungi and bacteria can degrade highly toxic compounds such as fluorinated pyrethroids, those found in agrochemicals [47,48].

Bacterial morphological diversity depends on genetic, environmental, and edaphic factors [34]. In this experiment, the differences in bacterial morphologies can partly be explained by variations in soil chemical properties (Table 2), as bacteria in these soils will vary depending on nutrient concentrations. Organic matter, nitrogen, and phosphorus influence similar bacterial morphologies, while soil texture does not significantly affect them [13,49]. The presence of structures such as spores in bacterial isolates from CM orchards may be partly explained by the higher manganese concentration found in these soils, as manganese is known to favor sporulation [50,51]. Although molecular identification of isolates to determine their genus and species was not performed in this study, it is highly likely that they belong to the most commonly reported genera in avocado rhizosphere: *Bacillus* and *Pseudomonas* [52,53].

The parameters evaluated to determine plant growth-promoting capacity through phosphorus and calcium solubilization, phosphatase activity, and nitrogen fixation have been used in other studies to assess the biological potential of soil bacterial isolates under various conditions [54]. Solórzano-Acosta et al. [55] found 13 bacterial isolates from avocado rhizosphere capable of phosphate solubilization and siderophore production. Among them, *B. subtilis* and *P. plecoglosicida* stimulated avocado seedling growth, increasing total dry biomass and leaf area. They also reported significant differences in nutrient absorption efficiency between inoculated and uninoculated plants. In this study, bacterial isolates from avocado orchards exhibited varying levels of plant growth-promoting activity. For phosphate solubilization, the highest number of solubilizing isolates was found in OM orchards. This may be because nutrient management in these plots does not involve inorganic phosphate fertilizers, prompting bacteria to activate solubilization mechanisms. This involves releasing organic acids like gluconic acid or 2-ketogluconic acid that chelate bioavailable phosphorus using hydroxyl and carboxyl radicals. The production of these acids varies depending on root exudates [56,57]. Additionally, OM orchards incorporate vermicompost, known to contribute bacterial diversity with phosphate-solubilizing activity [58]. These bacterial isolates also exhibited acid and alkaline phosphatase activity (APAA), indicating that they hydrolyzed esters and anhydrides of H_3_PO_4_, which are responsible for organic phosphorus mineralization and the release of inorganic phosphorus needed by plants [59,60]. These results are consistent with Luo et al. [61], who observed an average increase of 22–53% in acid and alkaline phosphatase activity compared to CM soils. The synthesis and activity of these enzymes are believed to be influenced by environmental pH [62,63]. Regarding calcium solubilization, as with phosphorus, the highest number of solubilizing isolates came from OM orchards. The application of organic amendments and leachates known to contain high calcium levels may explain the higher number of isolates found in organic vs. conventional management. Similar findings have been reported by Paredes-Mendoza and Espinosa-Victoria et al. [64] and Rana et al. [65], who isolated numerous calcium-solubilizing bacteria from soils amended with cattle manure. Biological nitrogen fixation by soil bacteria involves converting atmospheric inert nitrogen (N_2_) into ammonium (NH_4_^+^) or nitrate (NO_3_^−^), the forms plants can absorb and utilize [66,67]. This process is a sustainable alternative to nitrogen supply in crops. In this study, 100% of the bacterial isolates were capable of fixing atmospheric nitrogen, with OM isolates fixing about three times more nitrogen on average than CM isolates. Igiehon and Babalola [68] and Wolińska et al. [69] indicate that high organic matter content increases the populations of nitrogen-fixing bacteria in the form of nitrates. In contrast, the use of chemical nitrogen fertilizers (e.g., urea and ammonium nitrate), common in CM, can inhibit bacterial nitrogen fixation [70].

The plant growth-promoting capacity of rhizosphere bacteria from different plant species has been assessed to select candidates with high potential as biofertilizers [55]. In this study, all isolates were tested as growth promoters in tomato plants (*Solanum lycopersicum* M.). Fourteen isolates from OM orchards exhibited at least one growth-promoting mechanism, compared to only three from CM. Nitrogen fixation, calcium solubilization, and phosphate solubilization are among the various mechanisms by which bacteria enhance plant growth [71]. Most bacterial isolates in this study were able to produce nitrate, often contributing significantly to plant nitrogen supply [72]. Regarding P solubilization, only 25% of the total isolates exhibited variable solubilization capacity. Rodrigues et al. [73] found that ~47% of sugarcane endophytic bacteria showed phosphate solubilization, though at low levels. The greater number of phosphate-solubilizing isolates in OM orchards is likely due to the addition of microorganisms alongside organic fertilizers, enriching and altering microbial communities [74]. It is noteworthy that only one CM orchard isolate exhibited phosphate solubilization, indicating a high dependence on phosphate fertilization and low phosphorus availability in those soils. In this study, bacterial inoculation enhanced tomato seedling growth. Interestingly, some isolates that did not show growth-promoting activity still resulted in statistically similar growth to plants inoculated with active promoters. This suggests that some isolates may be using alternative mechanisms not assessed in this study, such as siderophore production or phytohormones like indole-3-acetic acid, known to promote plant growth [54,75].

The ability of microorganisms to absorb immobile nutrients like P and transfer them to host plants is a major benefit of microbial symbiosis. However, nutrient transfer capacity varies by microorganism [76]. Plant-microbe interactions influence nutrient transfer efficiency [77]. Thus, plant biomass production may vary depending on the microbial taxa colonizing roots due to their differing nutrient-supplying abilities, explaining the observed variation in tomato plant growth by bacterial isolate.

The antimicrobial activity of the isolates was determined through in vitro antagonism assays based on inhibition of the pathogen’s mycelial growth. The selection of bacterial isolates for their biocontrol capacity against agriculturally relevant pathogens has gained attention as a sustainable plant protection strategy [78,79]. In this study, 53% of bacterial isolates from CM orchards and 30% from OM orchards showed antagonistic effects against *Fusarium* sp., with results statistically similar to the positive control *B. subtilis*. Some soil bacteria are known to indirectly enhance plant growth by producing compounds that inhibit pathogens [80,81]. González-Sánchez et al. [82] reported that 22 isolates, mainly from the genera *Bacillus* and *Pseudomonas*, from healthy avocado tree roots and soils, exhibited antagonistic activity against *Rosellinia necatrix*, *Phytophthora cinnamomi*, *Phytophthora cactorum*, *Fusarium oxysporum*, *Rhizoctonia solani* and *Verticillium dahliae*. These genera have been widely reported as biocontrol agents against soil pathogens [83,84]. Their antifungal activity is attributed to the production of antifungal metabolites such as phenazine-1-carboxylic acid, phenazine-1-carboxamide, fengycin, surfactin, and bacillomycin [82]. Cortazar-Murillo et al. [85], working with two *Bacillus* rhizobacteria from avocado rhizospheres in Huatusco, Veracruz, and California, USA, evaluated their antagonistic activity against *Fusarium solani*, *Fusarium kuroshium* and *Phytophthora cinnamomi*. They found antimicrobial compounds in crude extracts and volatile emissions, including macrolactins, difficidin, bacillaene, and bacilysin, which inhibited pathogen growth by at least 20%. Similarly, Ruano-Rosa et al. [86], working with bacterial isolates from avocado rhizospheres in combination with *Trichoderma* strains for controlling root rot caused by *Rosellinia necatrix*, reported relative protective effects when applied individually. Combinations of *T. atroviride* with *P. chlororaphis* and *P. pseudoalcaligenes* strains showed better rot control, significantly reduced disease levels, and delayed symptom onset. Given the bacterial diversity and antagonistic results found in this study, it is likely that the bacterial isolates produce antimicrobial compounds responsible for their antagonistic activity. Some of these compounds could be of volatile nature, which have recently been shown to have antimicrobial activity in addition to promoting plant growth [87,88]. However, identifying the metabolites produced by these isolates is necessary to determine their nature and possible mechanisms of action.

## 5. Conclusions

The rhizospheric bacterial isolates obtained from *Persea americana* plots under conventional and organic management showed no differences in macroscopic or microscopic morphologies; however, differences in bacterial diversity were observed, with greater diversity in soils from *P. americana* plots under OM. This increase is likely due to the higher nutrient content in these soils. Regarding plant growth promotion, eight bacterial isolates from OM soils exhibited activity associated with plant growth promotion: phosphate solubilization, phosphatase activity, calcium solubilization, and biological nitrogen fixation. These bacterial isolates had a positive effect on the growth of *Solanum lycopersicum* and inhibited the growth of *Fusarium* sp. These results indicate that nutrient mineralization activity is higher in *P. americana* soils managed organically. Further characterization of these rhizospheric bacterial isolates could contribute to the development of innovative biofertilization products. The bacterial isolates characterized in this study are potential candidates for the formulation of bioinoculants for agriculturally important plants such as tomato and could also play a significant role in the biocontrol of phytopathogens. For future directions, we suggest exploring the integration of bacterial isolates with volatile compounds and evaluating their synergistic effects on plant growth and pathogen suppression, to advance biofertilizer and biocontrol formulations.

## Figures and Tables

**Table 1:** Physicochemical properties, number of bacterial isolates, and Shannon diversity index of avocado orchards under different agronomic management.

Physicochemical and Biological Parameters	Orchards with Conventional Management	Orchards with Organic Management
Textual Class	Sandy clay loam	Sandy loam
Bulk Density (g cm^−3^)	0.85	2
Porosity (%)	61.3	57.7
pH (H_2_O) 1:2	5.94	7.08
EC (dSm^−1^)	0.8	0.5
Organic Matter (%)	5.87	10.23
CEC meq100 g^−1^	20.65	40.5
Nitrogen (mg kg^−1^)	119.58	106.2
Phosphorus (mg kg^−1^)	169.17	330.8
Potassium (mg kg^−1^)	1827	1414
Calcium (mg kg^−1^)	1946	3688
Magnesium (mg kg^−1^)	689	1697
Sodium (mg kg^−1^)	23.2	265
Iron (mg kg^−1^)	64.8	56.9
Zinc (mg kg^−1^)	11.81	78.7
Manganese (mg kg^−1^)	42.4	39.23
Copper (mg kg^−1^)	20.5	29.26
Isolates bacterial	15 b ^1^	20 a
Shannon Diversity Index	1.75 (±0.24) b	2.44 (±0.08) a

^1^ Means with letters indicate significant differences (Student’s *t* test, *p* < 0.05). ±standard deviation. The mean of the isolates is the result of each of the subsamples (16), that made up the complete sample from each orchard.

**Table 2:** Physicochemical properties, number of bacterial isolates, and Shannon diversity index of avocado orchards under different agronomic management.

Morphological Characteristics of the Bacterial Isolate	Bacterial Isolates from Conventional Management Orchard Soils	Bacterial Isolates from Organic Management Orchard Soils
Colony size	Punctate, small, medium and large	Punctate, small, medium and large
Colony shape	Circular, fusiform, rhizoids and irregular	Circular, fusiform, rhizoids and irregular
Colony edge	Entire, undulate and rhizoids	Entire, rhizoids, filamentous and lobed
Colony transparency	Opaque and transparent	Opaque and transparent
Colony luster	L and WL *	L and WL
Colony color	White and yellow	White and yellow
Colony texture	Lisas	Lisas
Colony elevation	Convex, raised and flat	Raised and flat
Consistency	Soft, mucoid and hard	Soft, mucoid and hard
Positive Gram	8	10
Negative Gram	7	10
Microscopic morphology	Long bacilli with spores in initial, central and final positions	Long, short, wide, bacilli sporulated

* L: Luster; WL: Without Luster.

**Table 3:** Plant growth-promoting characteristics of bacterial isolates from orchards with different agronomic management.

Agronomic Management	Key to the Bacterial Isolate	Phosphate Solubilization Index	P-Nitrophenylphosphate Acids (µgmL^−1^)	P-Nitrophenylphosphate Alkaline (µgmL^−1^)	Calcium Solubilization Index	Nitrogen Fixation NO_3_^−^ ppm
	IBC-1	-	-	-	2.77 b	67 b
	IBC-2	-	-	-	-	64 b
	IBC-3	3.15 a *	15.87 a	8.48 b	-	73 b
	IBC-4	-	-	-	-	70 b
	IBC-5	-	-	-	-	71 b
Conventional Management	IBC-6	-	-	-	3.05 a	85.5 b
	IBC-7	-	-	-	-	96.75 b
	IBC-8	-	-	-	-	73 b
	IBC-9	-	-	-	-	74 b
	IBC-10	-	-	-	-	39 b
	IBC-11	-	-	-	-	63 b
	IBC-12	-	-	-	-	66 b
	IBC-13	-	-	-	-	73 b
	IBC-14	-	-	-	-	42 b
	IBC-15	-	-	-	-	70 b
	IBO-1	-	-	-	-	115 b
	IBO-2	-	-	-	-	57.7 b
	IBO-3	-	-	-	-	104 b
	IBO-4	-	-	-	-	157.5 a
	IBO-5	-	-	-	-	139.3 a
	IBO-6	-	-	-	-	139.3 a
	IBO-7	-	-	-	-	136.5 a
	IBO-8	-	-	-	-	72 b
	IBO-9	-	-	-	6.33 a	127.50 a
Organic	IBO-10	2.14 b	11.15 a	12.79 a	1.96 b	106.8 b
Management	IBO-11	-	-	-	-	117.50 b
	IBO-12	2.41 b	13.05 a	11.57 a	-	119.75 a
	IBO-13	3.5 a	15.88 a	13.76 a	2.83 b	162.5 a
	IBO-14	3.22 a	10.22 a	5.66 b	-	114 b
	IBO-15	-	-	-	-	123.8 a
	IBO-16	-	-	-	-	106.3 b
	IBO-17	4.31 a	14.64 a	11.91 a	4.06 a	125.5 a
	IBO-18	2.74 b	12.14 a	14.68 a	2.66 b	134.8 a
	IBO-19	2.95 b	13.03 a	6.56 b		132.5 a
	IBO-20	2.91 b	13.09 a	12.58 a	2.21 b	107.3 b
Positive control	*Bacillus thuringiensis*	2.46 b	0.345 b	3.01 b	2.93 b	-
	*Azotobacter vinelandii*	-	-	-	-	62.25 b

* Different letters within a column indicate a significant difference (Dunnett *p* < 0.05). The means were obtained from six repetitions. (-) = no activity. IBC: bacterial isolate from conventional plots; IBO: bacterial isolate from organic plots.

**Table 4:** Effect of inoculation with rhizospheric bacterial isolates from *P. americana* orchards under different agronomic management on the plant growth of *Solanum lycopersicum* under greenhouse conditions.

Agronomic Management	Key to the Bacterial Isolate	PS	CS	NF	PH cm	RL cm	TFB g	TDB g
	IBC-1	-	-	-	4.77 fg *	6.64 ab	0.238 c	0.065 b
	IBC-2	-	-	-	5.33 cd	6.02 b	0.150 d	0.098 b
	IBC-3	+	-	-	5.9 ab	4.05 g	0.270 cd	0.055 b
	IBC-4	-	-	-	4.42 h	6.33 b	0.271 c	0.047 bc
	IBC-5	-	-	-	4.78 fg	5.08 d	0.217 c	0.024 ef
Conventional Management	IBC-6	-	-	+	5.15 e	5.70 c	0.385 b	0.052 b
	IBC-7	-	-	-	5.09 ef	5.22 cd	0.250 c	0.041 c
	IBC-8	-	-	-	5.04 ef	4.09 fg	0.202 c	0.027 e
	IBC-9	-	-	-	5.05 ef	5.19 cd	0.174 cd	0.027 e
	IBC-10	-	-	-	5.65 b	4.6 e	0.234 c	0.033 d
	IBC-11	-	-	-	4.91 f	3.86 gh	0.229 c	0.032 d
	IBC-12	-	-	-	5.40 c	5.70 c	0.236 c	0.041 c
	IBC-13	-	-	-	4.59 gh	4.25 f	0.304 bc	0.053 b
	IBC-14	-	-	-	5.15 e	4.60 e	0.223 c	0.034 d
	IBC-15	-	-	-	6.33 ab	5.08 d	0.377 ab	0.061 b
	IBO-1	-	-	-	4.36 i	5.34 c	0.187 cd	0.035 d
	IBO-2	-	-	-	4.74 fg	6.91 ab	0.327 b	0.040 c
	IBO-3	-	-	-	5.13 e	5.76 c	0.218 c	0.032 d
	IBO-4	-	-	+	4.96 f	4.75 d	0.269 c	0.043 c
	IBO-5	-	-	+	4.79 fg	4.42 ef	0.272 c	0.056 b
	IBO-6	-	-	+	4.32 ij	5.49 c	0.275 c	0.048 b
	IBO-7	-	-	+	4.63 g	6.56 b	0.222 c	0.025 e
	IBO-8	-	-	-	4.75 fg	6.34 b	0.301 bc	0.069 b
	IBO-9	-	+	+	6.55 ab	5.51 c	0.316 b	0.054 b
Organic	IBO-10	+	+	-	5.89 ab	5.91 bc	0.443 a	0.062 b
Management	IBO-11	-	-	-	6.99 a	5.25 c	0.402 a	0.140 a
	IBO-12	+	+	+	6.6 a	4.65 d	0.344 b	0.138 a
	IBO-13	+	+	+	5.2 d	4.64 de	0.340 b	0.045 c
	IBO-14	+	+	-	5.44 b	6.06 b	0.224 c	0.108 a
	IBO-15	-	-	+	6.54 ab	7.95 a	0.324 b	0.032 d
	IBO-16	-	-	-	5.79 b	6.65 b	0.244 c	0.028 e
	IBO-17	+	+	+	5.70 b	6.28 b	0.402 a	0.041 c
	IBO-18	+	+	+	5.9 ab	7.12 a	0.348 b	0.026 e
	IBO-19	+	-	+	4.97 f	4.09 fg	0.246 c	0.039 c
	IBO-20	+	+	+	5.55 b	4.91 d	0.332 b	0.038 cd
Control	Without inoculation				3.65 j	3.19 h	0.141 d	0.018 f

* Different letters indicate a significant difference (Tukey *p* < 0.05). The means were obtained from twelve repetitions. (+): positive; (-): negative. IBC: bacterial isolate from conventional plots; IBO: bacterial isolate from organic plots. PS: phosphate solubilization; CS: calcium solubilization; NF: nitrogen fixation; PH: plant height; RL: root length; TFB: total fresh biomass; TDB: total dry biomass.

**Table 5:** Antagonistic activity of isolates from avocado orchards under different agronomic management practices against *Fusarium* sp. in vitro.

Agronomic Management	Key to the Bacterial Isolate	Percentage Inhibition of Micellar Growth *Fusarium* sp.
	IBC-1	-
	IBC-2	89.62 a *
	IBC-3	68.80 a
	IBC-4	88.97 a
	IBC-5	-
Conventional Management	IBC-6	86.28 a
	IBC-7	-
	IBC-8	92.35 a
	IBC-9	-
	IBC-10	76.44 a
	IBC-11	-
	IBC-12	-
	IBC-13	91.97 a
	IBC-14	92.33 a
	IBC-15	-
	IBO-1	-
	IBO-2	-
	IBO-3	93.69 a
	IBO-4	51.29 b
	IBO-5	-
	IBO-6	-
	IBO-7	-
	IBO-8	90.09 a
	IBO-9	69.51 a
Organic	IBO-10	-
Management	IBO-11	-
	IBO-12	-
	IBO-13	88.13 a
	IBO-14	-
	IBO-15	-
	IBO-16	-
	IBO-17	86.63 a
	IBO-18	-
	IBO-19	-
	IBO-20	-
Positive Control	*Bacillus subtilis*	79.06 a

* Different letters indicate a significant difference (Dunnett *p* < 0.05). The means were obtained from six repetitions. (-) = no activity. IBC: bacterial isolate from conventional plots. IBO: bacterial isolate from organic plots.

## Data Availability

The raw data supporting the conclusions of this article will be made available by the authors on request.

## Abbreviations

The following abbreviations are used in this manuscript:

CM	Conventional agronomic Management
OM	Organic agronomic Management
PGPB	Plant Growth-Promoting Bacteria
IBC	Isolates Bacterial from Conventional plots
IBO	Isolates Bacterial from Organic plots
PS	Phosphate Solubilization
CS	Calcium Solubilization
NF	Nitrogen Fixation
PH	Plant height
RL	Root Length
TFB	Total Fresh Biomass
TDB	Total Dry Biomass
